# Test-retest reliability of fMRI experiments during robot-assisted active and passive stepping

**DOI:** 10.1186/s12984-015-0097-2

**Published:** 2015-11-17

**Authors:** Lukas Jaeger, Laura Marchal-Crespo, Peter Wolf, Robert Riener, Spyros Kollias, Lars Michels

**Affiliations:** Department of Health Sciences and Technology, Sensory-Motor Systems (SMS) Lab, ETH Zurich, ML G 59, Sonneggstrasse 3, 8092 Zurich, Switzerland; Medical Faculty, University of Zurich, Zurich, Switzerland; Clinic of Neuroradiology, University Hospital of Zurich, Zurich, Switzerland; Center of MR-Research, University Children’s Hospital, Zurich, Switzerland

**Keywords:** fMRI, Test-retest, Reliability, Active, Passive, Stepping, Gait, Brain activation

## Abstract

**Background:**

Brain activity has been shown to undergo cortical and sub-cortical functional reorganisation over the course of gait rehabilitation in patients suffering from a spinal cord injury or a stroke. These changes however, have not been completely elucidated by neuroimaging to date, mainly due to the scarcity of long-term, follow-up investigations. The magnetic resonance imaging (MRI) compatible stepper MARCOS was specifically developed to enable the investigation of the supraspinal adaptations in paretic patients undergoing gait-rehabilitation in a controlled and repeatable manner. In view of future clinical research, the present study aims at examining the test-retest reliability of functional MRI (fMRI) experiments using MARCOS.

**Methods:**

The effect of repeated active and passive stepping movements on brain activity was investigated in 16 healthy participants from fMRI data collected in two separate imaging sessions six weeks apart. Root mean square errors (RMSE) were calculated for the metrics of motor performance. Regional overlap of brain activation between sessions, as well as an intra-class correlation coefficient (ICC) was computed from the single-subject and group activation maps for five regions of interest (ROI).

**Results:**

Data from eight participants had to be excluded due to excessive head motion. Reliability of motor performance was higher during passive than active movements, as seen in 4.5- to 13-fold lower RMSE for passive movements. In contrast, ICC ranged from 0.48 to 0.72 during passive movements and from 0.77 to 0.85 during active movements. Regional overlap of activations was also higher during active than during passive movements.

**Conclusion:**

These findings imply that an increased variability of motor performance during active movements of healthy participants may be associated with a stable neuronal activation pattern across repeated measurements. In contrast, a stable motor performance during passive movements may be accompanied by a confined reliability of brain activation across repeated measurements.

## Background

Exercises for functional gait rehabilitation, such as walking on a treadmill or with the aid of a robotic gait orthosis, have a major positive impact on restoration of walking in patients suffering from spinal cord injury or stroke. Previous studies investigating the effect of such functional gait-rehabilitation exercises on brain activity indicate a promotional effect for supraspinal plasticity in the motor centres expected to be involved in locomotion [1, 2]. However, the quality of this neural plasticity and its underlying physiological mechanisms have not been characterised in detail mainly due to the lack of standardised experimental conditions for follow-up studies. Longitudinal interventional studies combining extensive gait rehabilitation with a standardised controlled and measurable motor paradigm of the lower limbs during imaging of the brain might further disentangle the effect of gait training on brain activation. Newton et al. presented a motor paradigm of the lower limbs to investigate brain activity during simultaneous control of static moments around the hip, knee and ankle joint in one leg [3]. However, over ground walking involves the control of a dynamic and bilateral anti-phasic simultaneous movement of both legs under the transient influence of ground reaction forces. The magnetic resonance (MR) compatible stepper MARCOS has been developed to deliver and monitor repeated gait-like stepping movements in a standardised manner across task-related functional magnetic resonance imaging (fMRI) experiments [4]. The robot facilitates active (i.e. produced by the participant), as well as passive movements (i.e. imposed by the device). The investigation of passive movements can be meaningful in patients with no, or very limited, voluntary muscle activity in the legs as it is independent of performance ability, yet may be informative about the capacity for sensory adaptations to training [5]. Furthermore, the robot can impose loads against the soles of the feet along the cranio-caudal body axis mimicking ground reaction forces during stepping, thereby activating load-sensitive receptors in the lower limbs.

When investigating brain activation during lower limb motor control repeatedly over the period of rehabilitative intervention, knowledge on the test-retest reliability of the data is indispensable as activated brain areas have been shown to undergo test-retest effects between repeated imaging sessions [6]. In the context of interventional studies, information on the stability of a measurement serves as the basis for differentiating true effects caused by a therapy from those caused by variations in the experimental conditions.

In functional brain imaging, measures of reliability can be either calculated from single subject activation maps, or from activation maps derived from random effects group analyses, depending on whether conclusions shall be formulated for an individual participant or for a representative group of participants that was drawn from a particular population. In the context of interventional studies both are desirable, the former to judge on the effect of the intervention in a particular patient, the latter to generalise the findings of a study to a population.

A number of statistical tests have been proposed for judging the effects of repeated examination of brain activation. In motor control fMRI experiments in both healthy participants and patients, planned comparisons of activation maps, percent of signal intensity change, intra-class correlation coefficient (ICC), voxel count, overlap of activations between repeated sessions, coefficient of variation (CV) and the comparison of the location of the centre of gravity of activated clusters have been applied [3, 6–12]. Since all of these measures examine the retest-reliability of a given experiment from different perspectives, they have usually been combined to draw inferences.

An ICC, which is calculated from pairs of activation maps, appears to be the most appropriate measure of reliability for fMRI-data, since it is calculated from the variance components of the imaging data and does not depend on the magnitude of activations [13]. It has become the most widely used metric of reliability in fMRI studies.

Imaging studies using ICC as a measure of reliability of fMRI motor control experiments report in general good repeatability (values are deemed *excellent* above 0.75, *good* between 0.59 and 0.75, *fair* between 0.40 and 0.58 and *poor* for values below 0.40 [14]). Newton et al. reported the results of their study of unilateral single-joint lower limb motor control in which ICC was calculated from pairs of activation maps for two particular regions of interest (ROI). The voluntary production of torques led to individual ICC ranging from poor to excellent across subjects both in the primary sensorimotor (S1M1) and premotor (Brodmann Area (BA) 6) cortex [3]. In a recent reliability study investigating active and passive flexion and extension of the elbow using an MR-compatible manipulandum, fair to excellent ICC for active and passive movements was estimated for all of the investigated ROIs [11]. However, the test-retest reliability of a given paradigm and hence its ICC depends on numerous parameters throughout data acquisition and analysis, such as imaging hardware, resolution or spatial smoothing of the data (for a review see [15]). The comparability of different studies of reliability is therefore limited, and test-retest reliability needs to be established for each particular paradigm.

In view of future robot-aided fMRI assessments in longitudinal interventional studies, the aim of the present study was to assess the test-retest reliability of experiments using the robot MARCOS. The stability of motor performance and brain activation during the execution of two stepping conditions were investigated. Passive stepping without foot load and active stepping against a foot load of 40 % body weight were assumed to represent the most reliable and the least reliable motor task respectively. The passive condition was expected to yield more reliable results than the active condition, since the passive condition is robot-driven by a strict position control algorithm, while in the active condition motor behaviour is controlled by the participant, and therefore more variable.

## Methods

### Stepping robot

**Fig. 1:**
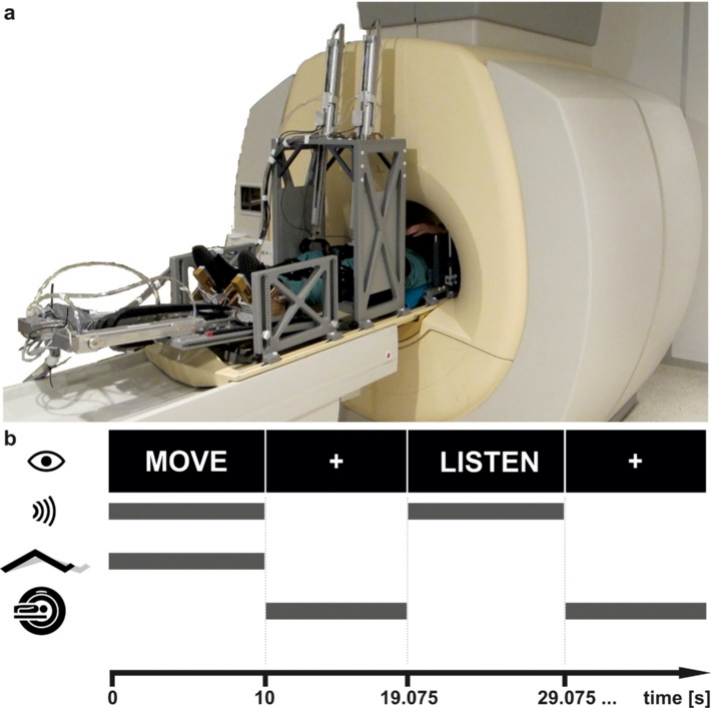
The investigational set-up as applied in the study. For the purpose of the present study, the MR-compatible robot MARCOS was mounted on the bench of the MR-scanner. The robot was used to measure and control the delivery of active and passive stepping movements (**a**). During task execution, the word “MOVE” was presented on the screen and participants conducted stepping movements in the rhythm of the concurrently presented auditory stimulus (metronome). This was followed by the acquisition of the BOLD-signal, while participants fixated on a white cross on the screen. Subsequently, the word “LISTEN” and the metronome were presented concurrently, again followed by the acquisition of the BOLD-signal (**b**)

The MR-compatible stepping robot MARCOS was employed to control active and passive stepping movements throughout the experiment [4, 16]. When placed inside the robot, the knees and feet of the participant are each attached to a pneumatic cylinder (Fig. 1a). The arrangement of the cylinders allows for one-degree of freedom flexion and individual extension movements of each leg, and the resulting gait-like stepping movement in the sagittal plane resembles “marching-on-the-spot”. In addition, external loads of up to 400 N along the cranio-caudal body axis can be rendered to the soles of the feet of the participants during movements by the foot cylinders in order to simulate ground reaction forces. The desired load at the foot is inversely proportional to the vertical position of the knee, such that highest force levels occur at full extension of the leg. Each of the four actuators is equipped with position and force sensors enabling accurate measurement of movement kinematics and kinetics. Data is sampled and stored at 80 Hz for off-line analysis of motor and robot performance. The robot is governed by two PCs: the sensor evaluation CPU runs on Linux and communicates via Ethernet with the control CPU running Matlab xPC real-time target. The control CPU executes control of the pneumatic valves as well as the control of the gait-pattern. Several redundant mechanisms were implemented in the robot to guarantee safety of the participants: 1) mechanical end stops prevent non-physiological postures of the lower limbs, 2) cylinder positions are measured, divergent positions cause the robot to shut down, 3) both CPUs are monitored by the control software and any error in the CPUs causes the robot to shut down, 4) watchdog circuits monitor the CPUs and the communication, 5) the operator can shut down the robot through emergency stops, and 6) upon an emergency stop, all cylinder chambers are set to atmospheric pressure and the cylinders can be moved freely.

A custom made hip and shoulder fixation as well as a custom made head bowl are combined with the inflatable Crania pillow (www.pearltec.ch) to prevent excessive task-related motion of the upper body and the head. MARCOS was built from materials of low magnetic susceptibility (i.e. aluminium, brass, polyvinyl chloride). MR-compatibility of the system was established by Hollnagel et al. [4].

### Experimental procedure

Sixteen healthy participants were investigated during two separate sessions (t_1_ and t_2_) six weeks apart. The chosen retest interval represents a common duration of rehabilitative gait interventions [17, 18], is in line with previous studies investigating the plasticity of brain activity in response to motor rehabilitation [1, 19] and corresponds to previous studies assessing reliability of fMRI signals [6, 11]. Participants were eligible for inclusion in the study if they did not meet any of the following exclusion criteria: 1) diagnosed neurological, musculoskeletal or cardiac dysfunction at present or in the past, 2) cardiac pacemaker, neuro-stimulator or hearing aid and 3) drug-abuse. The study was approved by the Ethics Committee of the Canton of Zurich (approval Nr. 856) and was conducted in accordance with the guidelines for research involving human subjects as outlined by the Declaration of Helsinki. All participants were informed about the aims and procedures of the study and gave their written consent for participation.

All participants performed passive stepping without foot load (*passive*) and active stepping with a load of 40 % of individual body weight acting against the foot soles (*active40*) in both imaging sessions. The leg movements of the participants (i.e. range of motion, stepping cadence and interaction forces with the robot) were measured online by the robot during both sessions. Movement conditions were tested in random order in separate runs of functional image acquisition in a blocked design. Each functional run consisted of 15 blocks of movement, interleaved with 15 blocks of a control condition. Each block lasted 10 s and was followed by 9.075 s of image acquisition. Movement cadence during both conditions was set to 0.5 Hz by the presentation of a metronome through the earphones [20, 21], yielding five steps per leg in each trial. Although the cadence was imposed by the robot during passive movements, the metronome was also presented in this condition, as well as during the control condition, to match auditory stimulation. The control condition served two purposes: firstly as a reference condition against which brain activity during movement trials was compared and, secondly, to control for auditory activations elicited by listening to the metronome per se. Visual cues were projected onto a screen near the feet of the participants at the start and for the duration of each block. Participants could see the screen by means of a mirror mounted to the head coil of the scanner. The word “MOVE” was presented for movement trials, while “LISTEN” was presented during control trials (Fig. 1b). As passive movements were imposed on the participants they were instructed to relax the muscles of their lower limbs and to not voluntarily contribute to flexion and extension of their lower limbs. During active movements, participants were instructed to voluntarily produce flexion and extension of their lower limbs in the rhythm set by the metronome. Under these conditions, the cylinders attached to the knees limited the range of motion and the cylinders attached to the feet rendered the desired foot loads as participants cycled through the steps. Furthermore, participants were instructed to fixate on a white cross on the screen during image acquisition between the “MOVE” and “LISTEN” blocks and to not rehearse or imagine movement execution when listening to the metronome alone. Participants were familiarised with each movement condition before the start of the experiment and informed about the upcoming type of movement before the start of each functional run.

### Image acquisition

Image acquisition from all participants was carried out on the same 1.5 T Philips Achieva scanner (Philips Medical Systems, Best, the Netherlands) at the University Hospital of Zurich using an 8-channel SENSE^TM^ head coil. The sparse sampling imaging protocol consisted of clusters of image acquisition interleaved with silent gaps of 10 s length [22]. Each imaging cluster comprised of three consecutive volumes (TR = 3.025 s). The duration between the onsets of two imaging clusters was hence 19.075 s. 93 volumes in 31 clusters of 3 volumes were acquired, using a whole brain T2*-weighted, single-shot, echo planar imaging (EPI) sequence (TE = 50 ms, flip angle = 90 °, SENSE factor = 1.6). 35 interleaved, angulated, transversal slices covering the whole brain were acquired in each volume (field of view = 220 mm × 220 mm, acquisition voxel size: 2.75 × 2.8 × 3.8 mm, resliced to 1.72 × 1.72 × 3.8 mm).

### Data analysis

#### Motor performance

Three metrics of motor performance were calculated for both stepping conditions at t_1_ and t_2_: *Knee amplitude* was defined as the range of motion of the knee per step and *stepping frequency* was defined as the number of steps of one leg per second. *Foot load* was defined as the maximal interaction force between the foot and the robot per step. Position and force data were extracted using custom Matlab routines (Matlab 2012b, Mathworks Inc., Natick, MA, USA, www.mathworks.com). Position data was filtered with a low pass 1st-order Butterworth filter with a cut-off frequency of 4 Hz and the mean *knee amplitude* and *stepping frequency* were extracted from each leg in each block of movement. The mean *foot load* was extracted from the data recorded from the force sensors at the foot cylinders per block. For each participant and condition, values were then averaged across all blocks. Data was further averaged over both legs, as *foot load*, *knee amplitude* and *stepping frequency* values of the left and the right leg were not significantly different (planned comparisons, all p-values > 0.1) in any of the conditions.

The left/right-averaged data of each performance metric of both sessions was then subject to an individual 2-way repeated measures ANOVA with the factors *time* and *condition*. This allowed testing for significant differences between time points and conditions. To test the hypothesis of no significant differences between t_1_ and t_2_ in all of the performance metrics within each condition, additional planned comparisons were applied in case of a significant main effect of *time*. The significance level for all statistical tests of motor performance was set to α = 0.05.

To further assess reliability of repeated test sessions, the root mean squared error (RMSE) of differences between t_1_ and t_2_ was calculated using the following formula:

$$ RMSE=\sqrt{\frac{{\displaystyle \sum_{i=1}^n{\left({x}_{2,i}-{x}_{1,i}\right)}^2}}{n}}, $$

where *n* denotes the total number of measurements of the metric at each session (i.e., 15) and *x*_1,*i*_& *x*_2,*i*_ are the *i* -th pair of values of the measurements at t_1_ and t_2_.The RMSE is an indicator of the absolute reliability. The absolute difference in measurements of the same metric repeated in two different sessions is expected to lie within 2.77*RMSE in 95 % of the measurements (normal distribution of the measurements is assumed, this was verified by means of visual inspection of Q-Q-plots) [23]. In other words, a true effect due to an intervention is likely if the difference between repeated measurements is higher than the RMSE multiplied by 2.77. Small RMSE values indicate low variability between measurements at t_1_ and t_2_. This indicator shall provide a practical of reliability for users of the robot.In order to compare the foot load variability to data in the literature, the CV was calculated across repeated measurement sessions using the following formula for each individual:

$$ CV=\left(\sqrt{\frac{{\displaystyle \sum_{i=1}^kS{D}_i^2*\left({n}_i-1\right)}}{{\displaystyle \sum_{i=1}^k{n}_i}-k}}/\frac{{\displaystyle \sum_{i=1}^kmea{n}_i}}{k}\right)*100, $$

where *mean*_*i*_ denotes the mean of measurement *i*, *SD*_*i*_ denotes the standard deviation of measurement *i*, *n*_*i*_ denotes the number of trials at measurement *i*, and *k* denotes the total number of measurements.

### Analysis of imaging data

All fMRI datasets were analysed using SPM8 (Wellcome Department of Cognitive Neurology, London, UK, www.fil.ion.ucl.ac.uk/spm) running on Matlab 2012b (Mathworks, Inc., Natick, MA, USA, www.mathworks.com). The first three volumes prior to the first task-block were removed from each run. In spatial preprocessing the remaining 90 volumes were firstly realigned to their mean image and unwarped to remove residual head motion related variance and image distortions along air-tissue boundaries [24]. Secondly, all data from t_2_ was coregistered to the mean image of the respective condition at t_1_. Thirdly, all images were normalised into standard MNI space using to the EPI-template provided by the Montreal Neurological Institute, re-sliced to a voxel size of 2 × 2 × 2 mm^3^, and finally all data was spatially smoothed (FWHM = 8 mm). The estimated realignment parameter data from the realignment step were filtered using the discrete cosine transform matrix filter (cut off at 128 s) incorporated in SPM8, to remove linear baseline drifts. Only data from participants whose estimated head motion parameters were below a stringent threshold of ½ voxel size after filtering in every spatial dimension in both conditions and at both experimental sessions were included in the subsequent statistical analysis. In the 1st-level analysis the data from t_1_ and t_2_ were modelled as two separate task regressors in the same general linear model (GLM) for each movement condition individually [25]. Two additional regressors were added to the model for each session to account for the T1-decay along consecutive volumes [26]. A high pass filter (cut off at 128 s) was used to remove slow signal drifts. To account for the sparse-sampling fMRI scheme, data taken during each trial was modelled using a boxcar function (1st-order, window length 3 x TR (i.e., 9.075 s)) [27]. Contrast images for each task regressor were calculated to reveal task-related activation at t_1_ and t_2_. To estimate the task-related effects at the group level, all contrast images of a specific task from the 1st-level analysis were subject to individual one-sample t-tests. Planned comparisons were computed, in order to test for significant differences between t_1_ and t_2_. The resulting activation maps were limited to a cluster-corrected voxel threshold of *p* < 0.001 (spatial extent: *k* ≥ 42 contiguous voxels) [28, 29]. The cluster threshold method was applied to control for the overall type I error. Anatomical correlates of clusters of activation were determined with the help of probabilistic cytoarchitectonic maps implemented in the Anatomy toolbox [30]. This toolbox was also used to define bilateral anatomical regions of interest (ROI) in the primary motor cortex (M1), primary somatosensory cortex (S1), secondary somatosensory cortex (S2) and the cerebellum Table 1. The ROI covering M1 was built by combining BA 4a and 4b [31]. BAs 1, 2, 3a and 3b served to create the ROI in S1 [32–34]. The ROI covering S2 was built by combining areas Operculum (OP) 1, OP2, OP3 and OP4 in the parietal operculum [35, 36]. The ROI located in the cerebellum was created by combining the lobules I to X (lobes and vermis) included in the Anatomy toolbox [37]. A ROI covering SMA was built from the automated anatomical labeling atlas [38] using the WFU_pickatlas toolbox [39]. These specific ROIs were selected as these areas have repeatedly been reported to be involved in lower limb motor control in previous studies [21, 22, 40–42].

### Indices of reliability

To assess the reliability of activations in these specific ROIs the following indices were calculated for the individual data at the 1st-level as well as for the 2nd-level group data from pairs of activation maps:

In order to gain insight into the spatial congruence of activations, the relative overlap of activations between t_1_ and t_2_ was determined by calculating the Sørensen-Dice index [43–45]:

$$ {R}_{overlap}=\frac{2*{V}_{overlap}}{V_1+{V}_2} $$

where *V*_*overlap*_ represents the number of voxels commonly activated at t_1_ and t_2_, and *V*_1_/ *V*_2_ represent the number of voxels that were activated at t_1_ or t_2_ respectively. This ratio of commonly activated voxels and the sum of activated voxels at the two sessions was calculated from activation maps that were limited to *p* ≤ 0.001, uncorrected for multiple comparisons. This index can range from 0 (no overlap) to 1 (perfect overlap) and is independent of the height of the t-values, once voxels have passed the threshold. However, a specific voxel with comparable activation at t_1_ and t_2_ might pass the threshold in one session, but only just fail to pass the threshold in the second session. In this case the denominator of the above ratio is increased, leading to an underestimation of the overlap between sessions.

To complement the results from *R*_*overlap*_, the ICC was calculated, a measure of reliability that is derived from activation maps without any statistical voxel threshold. A two-way mixed model for consistency between measurements, i.e. ICC(3,1) was applied in the current study [11, 46]. In the case of two repeated measurements, the ICC coefficient was calculated using:

$$ ICC\left(3,1\right)=\frac{BMS-EMS}{BMS+EMS} $$

where BMS denotes the Between voxel Mean Square variance, while EMS denotes the Error Mean Square variance. Using unthresholded data for the calculation of the ICC is legitimate, since the ICC is based purely on the variance of the data and does not depend on the level of activation itself. As such, voxels with low activation might exhibit high ICC coefficients, meaning they have consistent activation despite failing to pass significance in a *t*-test in the fMRI-analysis (i.e. in the case of voxels whose response to the stimulus poorly fits the modelled hemodynamic response function). However, at the same time the ICC might also include some voxels that were not involved in the task. The coefficient may range from 0 (low reliability) to 1 (perfect reliability). In the present study, ICCs were classified as *excellent* above 0.75, *good* between 0.59 and 0.75, *fair* between 0.40 and 0.58 and *poor* for values below 0.40, as proposed by [14].

The results calculated from the single subject data, R_overlap_ and ICC_single_ were then condensed by averaging across all participants during each movement condition, yielding mean values for R_overlap_ and ICC_single_. Fisher’s z-transform was applied to ICC_single_ values before averaging.

To further test for statistically significant differences of R_overlap_ or ICC_single_ across ROIs and conditions, the results of each reliability index derived from the 1st-level fMRI analyses were entered into a separate 2-way repeated measures ANOVA with the factors *condition* and *ROI*. To test the hypothesis of no significant differences within ROIs across conditions, additional planned comparisons were conducted in case of a significant main effect of condition (α = 0.05, with a Bonferroni correction for multiple comparisons). Normal distribution of the data was verified by visual inspection of Q-Q-plots.

## Results

All participants performed both stepping conditions at t_1_ and t_2_ during functional image acquisition. The retest interval between t_1_ and t_2_ ranged between 42 and 48 days. In both stepping conditions all data from 8 of the 16 participants was excluded from the analysis due to head motion exceeding ½ voxel size during image acquisition either at t_1_ or t_2_, or both. Most of the head motion occurred in the z-direction (inferior/superior), i.e. along the cranio-caudal body axis, probably reflecting the impact of the stepping movements of the legs. Characteristics of the study sample can be found in Table 2. The participants of the present study are a subset of those reported in [22].

**Table 1:** Definitions of regions of interest as used in the fMRI-analysis

Region of interest	Area
M1	BA 4a and 4b
S1	BA 1, 2, 3a & 3b
S2	OP1, OP2, OP3, OP4
SMA	SMA from AAL atlas
Cerebellum	Lobules I to X (Hemispheres and Vermis)

*M1* primary motor area, *S1* primary somatosensory area, *S2* secondary somatosensory area, *BA* brodmann area, *OP* operculum, *SMA* supplementary motor area, *AAL* automated anatomic labelling atlas [38]

**Table 2:** Anthropometric data of the final study sample

	Mean (SD)	min	max
Δt [days]	43 (2)	42	48
Age [years]	25 (1.9)	22	27
Body height [m]	171.4 (5.8)	165	181
Body weight [kg]	70.3 (8.5)	56.1	81.6
WHQ	15.25 (1.09)	13	16
WFQ	11 (6)	1	19

*Δt* days between session 1 and session 2, *WHQ* waterloo handedness questionnaire; values may range from −16 to 16, *WFQ* waterloo footedness questionnaire; values may range from −20 to 20, positive values represent dominance of the right side of the body in both tests, *SD* standard deviation

### Motor performance

The 2-way repeated measures ANOVA of the performance metric *knee amplitude* did not reveal a significant interaction effect between the factors of *time* and *condition* (F_1,7_ = 4.057, *p* = 0.084). The factor *condition* showed a significant main effect (F_1,7_ = 9.751. *p* = 0.017).

For the performance metric *foot force*, the 2-way repeated measures ANOVA did reveal a significant interaction effect between the factors *time* and *condition* (F_1,7_ = 6.083, *p* = 0.043). Furthermore, a significant main effect was found for both factors *time* (F_1,7_ = 9.974, *p* = 0.016) and *condition* (F_1,7_ = 419.307, *p* < 0.001). Planned comparisons for the factor *time* revealed a significant difference in the *foot force* between t_1_ and t_2_ in condition *active40* (T_7_ = 2.968, *p* = 0.021), but not in the condition *passive* (T_7_ = 0.615, *p* = 0.558) (Table 3).

**Table 3:** Motor performance during *passive* and *active40* stepping at session 1 (t_1_) and 2 (t_2_)

	Metric	t_1_	t_2_	*p*-value
Passive	Knee amplitude [m]	0.15 (0)	0.14 (0)	0.747
	Stepping frequency [Hz]	0.51 (0)	0.51 (0)	0.408
	Foot force [N]	47.41 (8.53)	46.29 (4.81)	0.558
Active40	Knee amplitude [m]	0.17 (0.02)	0.15 (0.02)	0.074
	Stepping frequency [Hz]	0.52 (0.02)	0.53 (0.02)	0.416
	Foot force [N]	246.20 (24.25)	231.23 (22.86)	**0.021**

Values are group means (standard deviation). The *p*-values denote significance of differences between means at t_1_ and t_2_ as assessed by planned comparisons. Significant results are highlighted in bold

Finally, the 2-way repeated measures ANOVA for the performance metric *stepping frequency* did not reveal a significant interaction between the factors *time* and *condition* (F_1,7_ = 0.554, *p* = 0.481). Furthermore, no significant main effect of *time* was detected (F_1,7_ = 0.957, *p* = 0.361). However, the factor *condition* showed a significant main effect (F_1,7_ = 12.805, *p* = 0.009).

**Fig. 2:**
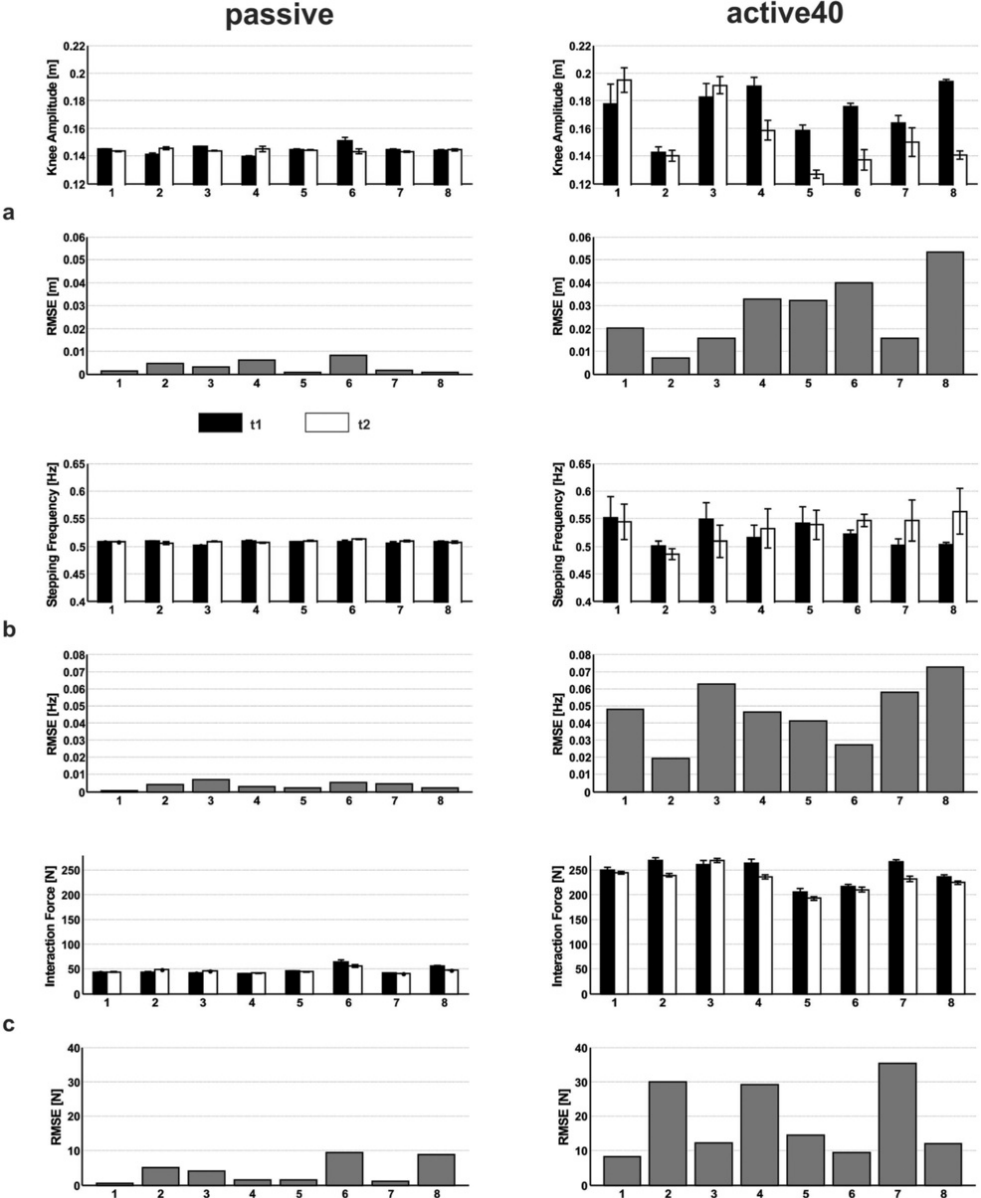
Motor performance and its reliability during passive and active40 stepping. Motor performance at session 1 (t1) and 2 (t2) and root mean squared error (RMSE) of differences between t1 and t2 of the individual participants during passive (*left column*) and active40 (*right column*) stepping. **a** knee amplitude, **b** stepping frequency and (**c**) foot force. Rows 1, 3, 5: mean ± one standard deviation at t_1_ and t_2_. Rows 2, 4, 6: RMSE of differences between t_1_ and t_2_

During *passive* stepping, the mean (standard deviation) RMSE of differences between t_1_ and t_2_ for *knee amplitude* was 0.0033 (0.0027) m, 0.0036 (0.002) Hz for *stepping frequency* and 3.9922 (3.5492) N for the *foot force*. During *active40* stepping, the mean RMSE of differences between t_1_ and t_2_ for *knee amplitude* was 0.027 (0.0152) m, 0.047 (0.0177) Hz for *stepping frequency* and 18.8534 (10.7748) N for *foot force* (Fig. 2). When comparing the RMSE values between the two movement conditions, values were about 8-fold higher for *knee amplitude*, about 13-fold higher for *stepping frequency* and 4.5-fold higher for *foot force* in the condition *active40* than in *passive*. The higher RMSE for *foot force* in *active40* is supported by a higher mean CV of 2.21 (0.45) % for *active40* vs. 1.83 (1.94) % in *passive*.

### Functional brain activation during stepping at t_1_ and t_2_

**Fig. 3:**
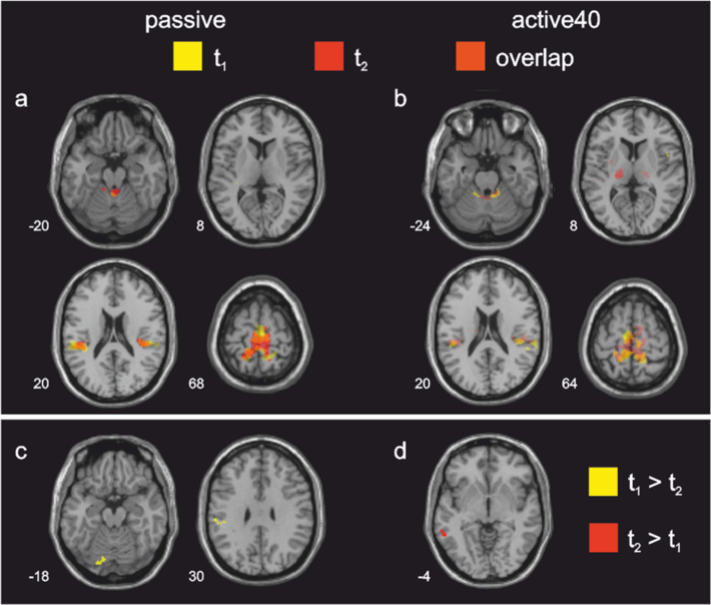
Activation maps during passive and active40 stepping. *Top row*: Regions of significant BOLD signal increase during passive (**a**) and active40 (**b**) stepping at session 1 (t_1_) and 2 (t_2_), and their overlap. *Bottom row*: Areas of significantly higher BOLD signal increase at either t_1_ or t_2_ for passive (**c**) and active40 (**d**). Time between t_1_ and t_2_ ranged between 42 and 48 days. The sections were taken at the z-coordinate indicated at the bottom left of each section, images are displayed in neurological convention (i.e., left is left), *p* ≤ 0.001, cluster corrected, k = 42 voxels

During *passive* stepping, overlapping activation across t_1_ and t_2_ was found in bilateral S1/M1, superior parietal lobe, S2, SMA proper and the cerebellar vermis. At both measurement sessions the middle cingulate gyrus was furthermore activated, albeit these clusters did not spatially overlap. During *active40* stepping, overlapping cortical activation across t_1_ and t_2_ was found in bilateral S1/M1, superior parietal lobe, S2 and SMA proper. Overlapping subcortical activation in the anterior and posterior cerebellar vermis was furthermore found in this condition. At t_2_ bilateral activation of the thalamus was additionally found during *active40* (Table 4, Fig. 3a and b).

**Table 4:** Cortical and sub-cortical regions of significant BOLD signal increase during *passive* and *active40* stepping

		Left hemisphere							Right hemisphere						
		Region	Area	T	k_E_	x	y	z	Region	Area	T	k_E_	x	y	z
Passive	t1	S2	OP1	18.395	448	−52	−30	18	Supramarginal Gyrus		14.071	406	50	−32	34
		Precuneus		17.097	1142	−14	−46	56	-		-	-	-	-	-
		Vermis	I-IV	10.553	164	−2	−52	−6	-		-	-	-	-	-
		Middle Cingulate Gyrus	CMA	7.835	44	−8	−12	46	-		-	-	-	-	-
		-		-	-	-	-	-	Lingual Gyrus		9.831	147	2	−82	−10
	t2	Vermis	I-II	12.676	126	−6	−48	−28	-		-	-	-	-	-
		-		-	-	-	-	-	Paracentral Lobule		12.053	2103	8	−38	60
		S2	OP1	10.189	127	−46	−30	18	Supramarginal Gyrus		8.769	221	56	−30	28
		Cerebellum	I-IV	7.832	62	−22	−32	−30	-		-	-	-	-	-
Active40	t1	SMA	BA6	14.444	1139	−6	−16	66	-		-	-	-	-	-
		Posterior Vermis	VIIIa	10.826	92	−2	−76	−42	-		-	-	-	-	-
		-		-	-	-	-	-	Insula		8.888	91	50	10	0
		Anterior Cerebellum	I-IV	10.814	50	−24	−32	−36	Anterior Cerebellum	I-IV	14.652	300	16	−38	−26
		S2	OP1	9.815	258	−60	−22	14	S2	OP1	7.091	175	44	−30	20
	t2	M1	BA4	30.251	2474	−14	−40	58	-		-	-	-	-	-
		Thalamus	lateral posterior nucleus	17.106	325	−18	−22	10	Thalamus	ventral posterior lateral nucleus	8.593	124	22	−22	2
		S2	OP1	11.93	225	−40	−26	16	Supramarginal Gyrus		14.584	237	62	−18	28
		Vermis	VIIIb	11.194	87	−2	−64	−40	Vermis	I-IV	11.474	325	2	−44	−20
		Insula		9.284	52	−36	4	16	-		-	-	-	-	-

Coordinates indicate the location of the peak activation in each cluster. All coordinates are in MNI-space, voxel threshold was ***p*** ≤ 0.001, cluster-corrected, *k* = 42 voxels

*S2* secondary somatosensory cortex, *SMA* supplementary motor area, *S1/M1* primary sensorimotor cortex, *CMA* cingulate motor area

### Repeatability of fMRI measurements

Planned comparisons between the activation maps at t_1_ and t_2_ for *passive* and *active40* stepping revealed only a minor, yet significant, difference between measurements in both movement conditions. When compared to t_2_, *passive* stepping at t_1_ led to significantly higher activation in the left supramarginal gyrus and in the cerebellar vermis (Fig. 3c). No area showed significantly higher activation at t_2_ than at t_1_ in the *passive* condition. During *active40* stepping at t_2_, significantly higher activation was only found in the left middle temporal sulcus than at t_1_ (Fig. 3d), while no area showed significantly higher activation at t_1_ than at t_2_ during *active40*.

These small group-level differences between measurements at t_1_ and t_2_ in both stepping conditions are not fully supported by the ROI-analysis. For *passive* stepping, only a small amount of overlapping activation between 2nd-level group data at t_1_ and t_2_ was found in the cerebellum and S2, while M1, S1 and SMA lacked any overlapping activation (i.e., R_overlap_ = 0). For *active40* stepping, overlapping activation was found in all of the investigated ROIs (Table 5).

**Table 5:** Single subject and group values of R_overlap_ and ICC in each region of interest

		R_overlap_					ICC				
		Cerebellum	M1	S1	S2	SMA	Cerebellum	M1	S1	S2	SMA
Passive	Single	0.17 (0–0.38)	0.67 (0.16–0.9)	0.43 (0–0.68)	0.51 (0.06–0.86)	0.57 (0–0.8)	0.45 (0.24–0.69)	0.88 (0.7–0.93)	0.73 (0.61–0.83)	0.75 (0.56–0.87)	0.77 (0.43–0.9)
	Group	0.32	0	0	0.4	0	0.7	0.72	0.48	0.63	0.5
Active40	Single	0.44 (0.16–0.6)	0.69 (0.23–0.92)	0.39 (0.04–0.55)	0.5 (0.1–0.73)	0.64 (0.25–0.84)	0.7 (0.53–0.77)	0.84 (0.61–0.91)	0.7 (0.57–0.83)	0.74 (0.6–0.86)	0.83 (0.68–0.91)
	Group	0.62	0.4	0.23	0.52	0.2	0.85	0.8	0.81	0.85	0.77

single: values are mean (minimum to maximum) calculated from the results of 1st-level single subject analyses at t_1_ and t_2_, group: values were calculated from results of the 2nd-level group analysis at t_1_ and t_2_. M1 = primary motor cortex, S1 = primary somatosensory cortex, S2 = secondary somatosensory cortex, SMA = supplementary motor area

Furthermore, the ROI analysis revealed higher ICCs for activations during *active40* than during *passive* stepping in the cerebellum, S1, S2 and SMA, but not M1. ICC_group_ calculated from the 2nd-level group data during *passive* stepping revealed fair repeatability for S1 and SMA and good repeatability for S2, the cerebellum and M1. During *active40* stepping excellent repeatability was found for all of the ROIs (Table 4).

**Fig. 4:**
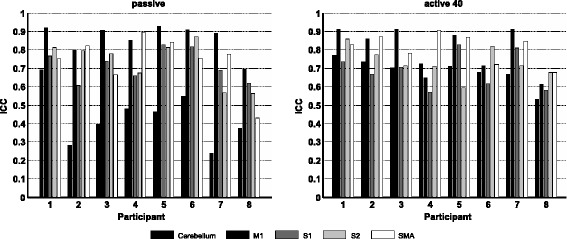
Individual test-retest reliability of brain activation in the investigated regions of interest. Reliability of individual activations (ICCsingle) is given for the regions of interest (ROI) cerebellum, M1, S1, S2 and SMA between t1 and t2. Some participants demonstrate consistently higher ICC than others. T-values were extracted from each ROI and reliability was assessed during passive (*left*) and active 40 (*right*) stepping. M1 = primary motor cortex, S1 = primary somatosensory cortex, S2 = secondary somatosensory cortex, SMA = supplementary motor area

When calculating average repeatability from the individual 1st-level fMRI data during the *passive* condition, fair ICC_single_ was found for the cerebellum, good ICC_single_ for S1 and excellent ICC_single_ for M1, S2 and SMA. During *active40* stepping good reliability of activations was found in the cerebellum, S1 and S2, while excellent averaged ICC_single_ was found in M1 and SMA (Fig. **4**). Individual ICC_single_ during *passive* stepping ranged from poor to good in the cerebellum and from fair to excellent in S2 and SMA, while in M1 and S1, ICC_single_ ranged from good to excellent. During *active40* stepping ICC_single_ ranged from fair to excellent in the cerebellum and S1 and from good to excellent M1, S2 and SMA (Table 5).

The 2-way repeated measures ANOVA for ICC_single_ scores revealed a significant interaction effect of *condition* by *ROI* (F_4,28_ = 16.173, *p* < 0.001). Furthermore, there was a significant main effect of *ROI* (F_4,28_ = 16.923, *p* < 0.001), but no significant main effect of *condition* (F_1,7_ = 3.538, *p* = 0.101).

The 2-way repeated measures ANOVA for the single subject R_overlap_ scores revealed a significant interaction effect of *condition* and *ROI* (F_4,28_ = 3.537, *p* = 0.019). Furthermore, there was a significant main effect of *ROI* (F_4,28_ = 13.206, *p* < 0.001), but no significant main effect of *condition* (F_1,7_ = 0.413, *p* = 0.541).

## Discussion

The present study explored the test-retest reliability of motor performance and brain activation of a novel robot-aided experimental fMRI paradigm at the individual and group-level. The consistency of task-induced blood oxygenated level dependent (BOLD)-signal was compared between repeated measurements of active and passive gait-like stepping in the MR-compatible stepper MARCOS. To the authors’ knowledge, this is the first fMRI reliability study of brain activation during bilateral multi-joint lower limb movements.

### Motor performance

*Passive* stepping did not reveal any statistically significant differences in motor performance between the measurements at t_1_ and t_2_. The absence of statistically significant differences indicates very stable performance by the robot across repeated measurement sessions during *passive* movements. High repeatability during this condition is further supported by a low RMSE between t_1_ and t_2_ for each of the three metrics. Since the robot was strictly governed by position control in this condition, it was expected that the performance metrics would exhibit very low variability.

The healthy participants in this study reported in general no difficulties in maintaining limb passivity during the movements that were imposed by the robot. This observation is supported by low values of *foot force* and negative interaction forces between the robot and the participants at the knees (not shown), meaning that their legs were indeed suspended by the fixations during passive steps. High interaction forces would indicate a lack of muscle relaxation. In experiments with neurologically impaired patients this could, for example, point towards the presence of spasticity in the involved muscles, i.e. an increased resistance to imposed movement. The low RMSE for *foot force* further indicates that the participants were able to maintain limb passivity at similar levels during both experiments.

The metric *foot force* was significantly smaller at t_2_ than at t_1_ during *active40* stepping. However, the CV of the *foot force* during *active40* in the present study is smaller than the CV of the vertical ground reaction force during ground level walking (7 % in [47], single subject measured 9 times over 3 days), or during walking on the treadmill (5–8 % in [48], 10 subjects over 40 steps). Therefore this difference may be attributed to the variability inherent in the human motor system and interpret this finding as not physiologically relevant. This rationale is furthermore supported by the fact that no influence of movement performance on activity in specific regions of the brain was found in previous work [22]. The smaller *foot forces* at t_2_ were driven by the concurrent reduction in *knee amplitude*, since in *active40* stepping the robot is governed by a controller generating foot forces in proportion to the position of the knees. Individual RMSE between t_1_ and t_2_ of all motor performance metrics were 4.5 to 13-fold above those during *passive* stepping, indicating higher variability of movements between measurements at t_1_ and t_2_ in *active40*. High RMSE-values also indicate a limited sensitivity of the experimental set-up in this condition. In the context of an interventional study, it would hence be less likely to detect an actual rehabilitation related change in the motor performance during active movements (e.g. a reduction in movement variability).

The stepping frequency of 0.5 Hz investigated in the present study was approximately three times lower than that of over-ground gait at a comfortable speed in healthy participants (approximately 1.75 Hz [47]). In view of future clinical work with neurological patients, a reduced stepping frequency was chosen for two reasons: Firstly, lower stepping frequencies induce less task-related head motion (results from pilot experiments not shown), a factor that positively contributes to data quality. Secondly, lower stepping frequencies are well tolerated by neurological patients who are prone to developing spasticity, in particular during passive movements (results from pilot experiments not shown).

### Activated areas during stepping

Both stepping conditions led to significant BOLD signal increases at t_1_ and t_2,_ as compared to the control condition, in areas which have been previously reported to be involved in supine gait-like stepping [22], in pedaling [21, 40, 41], as well as during isolated movements of the lower limbs [42, 49].

### Reliability of fMRI measurements at the group level

After the analysis of the estimated head motion parameters from the realignment step, eight participants had to be excluded from both stepping conditions. This high dropout rate exemplifies that the test-retest reliability of the presented paradigm is a priori and is limited by the task-induced head motion in many participants. This occurred despite extensive body fixation applied to the participants at the torso and the head.

In those participants included in the analysis, the comparison of activations elicited by *passive* stepping at t_1_ and t_2_ revealed only minor, yet significant, differences, accompanied by minor and statistically non-significant changes in motor performance. They specifically consisted of higher activity observed in the left supramarginal gyrus and the cerebellar vermis at t_1_. Participants were able to maintain limb passivity at equal levels during both sessions. However, many of the participants reported that passiveness of the limbs required considerable concentration and attention to the task. Due to the novelty and relatively unnatural character of the task at t_1_, imposed passivity might have caused a higher cognitive load than at t_2_, and this might be reflected by session-specific variations in the related cortical processes causing differences in test-retest outcome measurements. The supramarginal gyrus has been shown to be involved in motor attention [50], hence its differential activation between sessions may indicate an effect of habituation from t_1_ to t_2_, despite the provision of rehearsal trials before image acquisition of each movement condition. Differences between activations induced by repetition of the same motor task have also been reported by Loubinoux et al. These authors argued that reduced levels of stress, arousal and attention may contribute to the differences between repeated measurements, as the component of novelty is attenuated in a second session [6]. Reductions in activation have also been associated with motor learning [51]. However, the design of the current study did not include a motor learning component. Regarding the activation differences between sessions it must also be noted that some signal clusters are located in the vicinity of the cerebellar tentorium, a region of the brain that is susceptible to motion artifacts due to the tissue boundaries in this area [52, 53].

The findings of R_overlap_, the index assessing the overlap of activations between sessions at t_1_ and t_2_, only partially support the findings from the voxel-wise whole group brain analysis. When calculating this metric from the group data, congruent activation during *passive* stepping was found in the cerebellum and S2, but not in any of the other ROIs. Overlapping activation in the paracentral lobule (including the medial aspects of M1 and S1) and SMA were found when thresholding the group t-maps during *passive* stepping more liberally at *p* ≤ 0.005 1(uncorrected). Cáceres et al. emphasised that high variability in the data together with the low number of participants, as it often is the case in neuroimaging experiments, hinders the power to detect effects [13]. Therefore, with the inclusion of a higher number of participants in *passive* stepping, an overlap of activation in these areas between t_1_ and t_2_ at the group level might be demonstrated at the current threshold.

Intriguingly, group R_overlap_ was consistently, yet not significantly, lower during *passive* than during *active40* movements in all of the investigated ROIs. Yet, this is in line with the fact that fewer significant differences between t_1_ and t_2_ were found at the whole brain level during *active40* than *passive* stepping.

**Fig. 5:**
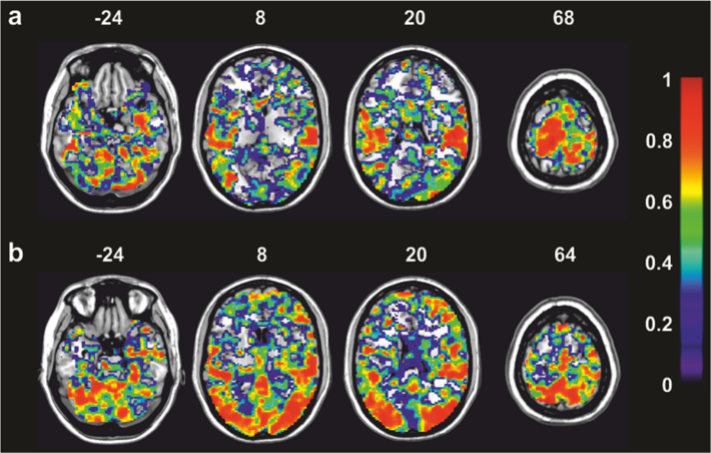
Voxel-wise maps of intra-class correlation coefficients for repeated sessions of passive and active40 stepping. Maps of intra-class correlation coefficients (ICCgroup) for repeated sessions of passive (**a**) and active40 (**b**) stepping shown on different axial slices (the z-coordinate is indicated at the top of each slice). Bilateral S2 and the paracentral lobule show high ICC in both conditions, while occipital, posterior parietal and prefrontal regions show high ICC as well in active40. Areas with high ICC (*red*) are hence not necessarily congruent with areas of activation above threshold. Images are displayed in neurological convention (i.e. left is left). The scale on the right indicates the ICC

The low congruence of activations during repeated *passive* stepping is in line with the values of ICC_group_ in these ROIs (i.e. fair to good). This indicates that the activations elicited by *passive* stepping in healthy participants are not overly reliable in S1 and SMA, but more robust in the cerebellum, M1 and S2, if a measurement is repeated after several weeks. In the latter three ROIs the activation of voxels was hence on similar levels at t_1_ and t_2_ (good ICC_group_), but did not reach significance (*p* ≤ 0.001, uncorrected) at the group level at either one or both measurement sessions, and hence R_overlap_ was low in these areas. This reflects the fact that voxels can have stable signals across sessions leading to good ICC but at the same time do not necessarily fit the HRF model very well as reflected by low t-values (Fig. **5**) [13].

Despite the differences and higher variability of motor behaviour between sessions i.e. higher RMSE values of kinematic metrics during *active40* stepping, widespread differences on the side of the supraspinal activations were absent, except for one small cluster of 55 voxels in the left posterior middle temporal lobe. The statistical comparison of activation maps at the whole brain level implicates that the generation and control of active movement induces more robust and consistent neural activation across sessions than the monitoring of passive movements. This is supported by the indices of test-retest reliability of fMRI measurements that were computed from the 2nd-level group data. Higher levels of overlap (R_overlap_) between activations at t_1_ and t_2_ were found during *active40* than during *passive* stepping in all of the investigated ROIs. This finding is accompanied by excellent values of ICC_group_ in all of the ROIs. The observation that activations were generally more robust during active than during passive movements is underpinned by a previous study of robot-assisted unilateral elbow movements [11]. The values of R_overlap_ and ICC reported by Estévez et al. were slightly, yet consistently, higher across investigated ROIs for active than for passive movements regardless of whether the values were calculated from 1st-level or 2nd-level data. In contrast, Loubinoux et al. did not find differences in the reliability of activations between active and passive movements [6].

### Reliability of fMRI measurements at the single subject level

In contrast to a complete lack of overlapping activations during *passive* movements in three of the five ROIs (M1, S1 and SMA) at the group level, R_overlap_ was different from zero in all of the ROIs at the single subject level. However, mean values were lower than those reported by [11]. The ranges of ICC_single_ in the present study are slightly above those of the unilateral single-joint lower limb motor control experiment using ICC as an indicator of reliability by Newton et al. [3]. The comparison of ICCs from Newton et al. with those of the present study indicates that test-retest reliability can be increased to some extent by the use of a robot to standardise the motor task between participants. Mean values of ICC_single_ are in the realm of those reported by Estévez et al. who also applied a robotic device to control and measure movements [11].

Considerable variability in both reliability indices calculated from pairs of individual t-maps (R_overlap_ and ICC_single_, Table 5) was found in this study. This finding is compatible with a study of Wei et al., who reported that between subject variance is higher than within subject variance in fMRI experiments [54], a finding that was also reported by [10]. This group found that variation in imaging data can be largely explained by differences in the signal-to-noise ratio (SNR) between individual measurements and that good ICC is achieved if the SNR of a particular measurement alone is high [10]. In the present study ICC_single_ was on a similar level in most participants across ROIs (except for participant 8 who had consistently lower values in all ROIs, Fig. **4**). Surprisingly, the SNR of this participant was not lower than that of the other participants (not shown).

### Potential implications for patient studies

Several groups have evaluated the test-retest reliability of fMRI experiments in stroke patients. Kimberley et al. found that stroke patients had somewhat higher ICC of fMRI results than healthy controls in a drawing task [8], while Eaton et al. reported approximately equal reliability between aphasic stroke patients and healthy controls in a language task [55]. However, the study of Kimberley et al. investigated repeatability using the unaffected hand. It can therefore only be speculated about test-retest reliability of experiments involving the paretic side of the body. Kimberley et al. discussed the possibility that increased between-subject variability artificially heightened their measures of reliability. Variability between study subjects may be increased in stroke patients due to heterogeneity in the study sample with regards to time since stroke, extent of recovery or lesion size and location.

The finding that reliability in stroke patients is comparable to that of healthy controls is somewhat surprising, since factors such as increased head motion [56] and age of patients [57, 58] may decrease the SNR and in turn the reliability. As the mean age of stroke patients is usually higher than that of the healthy participants in the present study, test-retest reliability of the presented paradigm could hence be lower when applied to a stroke cohort. Huettel et al. suggested to ameliorate the limitation of decreased SNR in the elderly by increasing the number of trials [57]. However, increasing the number of trials, and thereby the length of the fMRI experiment, will in turn very likely increase head motion, and this may again, to some extent, cancel out the gain in SNR. There is, therefore, a large potential in the combination of MARCOS with prospective motion correction during BOLD signal acquisition. These systems capture the movement of the head during an experimental run and adjust the pulse sequence in real-time such that the field-of-view remains in alignment with the brain tissue (for a review see [59]).

## Conclusions

The results of the present study in healthy participants indicate that activations during passive movements are less robust over repeated measurement sessions than those during active movements despite lower variability of motor performance during passive movements. The high variability of ICC_single_ between individual participants during both movement conditions renders the presented approach less suitable for making inferences at the single-subject level. The fact that half of the participants had to be excluded from image analysis due to excessive task-induced head motion implies a limited feasibility for studies with patients. The group results from the remaining participants, however, revealed fair to excellent test-retest reliability. This implies feasibility of the method for studies investigating basic neurophysiological principles and to draw conclusions that can be generalised to the populations from which the study participants were selected.

## References

[1] Winchester P, McColl R, Querry R, Foreman N, Mosby J, Tansey K (2005). Changes in supraspinal activation patterns following robotic locomotor therapy in motor-incomplete spinal cord injury. Neurorehabil Neural Repair.

[2] Luft AR, Macko RF, Forrester LW, Villagra F, Ivey F, Sorkin JD et al. Treadmill exercise activates subcortical neural networks and improves walking after stroke a randomized controlled trial. Stroke. 2008;39(12):3341–50. doi: 10.1161/Strokeaha.108.527531. 10.1161/STROKEAHA.108.527531 PMC2929142 18757284

[3] Newton JM, Dong Y, Hidler J, Plummer-D'Amato P, Marehbian J, Albistegui-DuBois RM (2008). Reliable assessment of lower limb motor representations with fMRI: Use of a novel MR compatible device for real-time monitoring of ankle, knee and hip torques. Neuroimage.

[4] Hollnagel C, Brügger M, Vallery H, Wolf P, Dietz V, Kollias S (2011). Brain activity during stepping: A novel MRI-compatible device. J Neurosci Methods.

[5] Dobkin BH, Firestine A, West M, Saremi K, Woods R (2004). Ankle dorsiflexion as an fMRI paradigm to assay motor control for walking during rehabilitation. Neuroimage.

[6] Loubinoux I, Carel C, Alary F, Boulanouar K, Viallard G, Manelfe C (2001). Within-session and between-session reproducibility of cerebral sensorimotor activation: a test-retest effect evidenced with functional magnetic resonance imaging. J Cereb Blood Flow Metab.

[7] Alkadhi H, Crelier GR, Boendermaker SH, Golay X, Hepp-Reymond M-C, Kollias SS (2002). Reproducibility of primary motor cortex somatotopy under controlled conditions. Am J Neuroradiol.

[8] Kimberley T, Khandekar G, Borich M (2008). fMRI reliability in subjects with stroke. Exp Brain Res.

[9] Kimberley T, Birkholz DD, Hancock RA, VonBank SM, Werth TN (2008). Reliability of fMRI during a continuous motor task: assessment of analysis techniques. J Neuroimaging.

[10] Raemaekers M, Vink M, Zandbelt B, van Wezel RJA, Kahn RS, Ramsey NF (2007). Test–retest reliability of fMRI activation during prosaccades and antisaccades. Neuroimage.

[11] Estévez N, Yu N, Brügger M, Villiger M, Hepp-Reymond M-C, Riener R (2014). A reliability study on brain activation during active and passive arm movements supported by an mri-compatible robot. Brain Topogr.

[12] Havel P, Braun B, Rau S, Tonn JC, Fesl G, Brückmann H (2006). Reproducibility of activation in four motor paradigms. J Neurol.

[13] Caceres A, Hall DL, Zelaya FO, Williams SCR, Mehta MA. Measuring fMRI reliability with the intra-class correlation coefficient. Neuroimage. 2009;45(3):758–68. http://dx.doi.org/10.1016/j.neuroimage.2008.12.035. 10.1016/j.neuroimage.2008.12.035 19166942

[14] Cicchetti DV, Sparrow SA (1981). Developing criteria for establishing interrater reliability of specific items: applications to assessment of adaptive behavior. Am J Ment Defic.

[15] Bennett CM, Miller MB (2010). How reliable are the results from functional magnetic resonance imaging?. Ann N Y Acad Sci.

[16] Hollnagel C, Vallery H, Schädler R, López I-L, Jaeger L, Wolf P (2013). Non-linear adaptive controllers for an over-actuated pneumatic MR-compatible stepper. Med Biol Eng Comput.

[17] Werner C, Von Frankenberg S, Treig T, Konrad M, Hesse S (2002). Treadmill training with partial body weight support and an electromechanical gait trainer for restoration of gait in subacute stroke patients: a randomized crossover study. Stroke.

[18] Mayr A, Kofler M, Quirbach E, Matzak H, Fröhlich K, Saltuari L (2007). Prospective, blinded, randomized crossover study of gait rehabilitation in stroke patients using the Lokomat Gait Orthosis. Neurorehabil Neural Repair.

[19] Miyai I, Yagura H, Hatakenaka M, Oda I, Konishi I, Kubota K (2003). Longitudinal optical imaging study for locomotor recovery after stroke. Stroke.

[20] Ciccarelli O, Toosy A, Marsden J, Wheeler-Kingshott C, Sahyoun C, Matthews P (2005). Identifying brain regions for integrative sensorimotor processing with ankle movements. Exp Brain Res.

[21] Mehta JP, Verber MD, Wieser JA, Schmit BD, Schindler-Ivens SM (2009). A novel technique for examining human brain activity associated with pedaling using fMRI. J Neurosci Methods.

[22] Jaeger L, Marchal-Crespo L, Wolf P, Riener R, Michels L, Kollias S (2014). Brain activation associated with active and passive lower limb stepping. Front Hum Neurosci.

[23] Bland JM, Altman DG (1996). Measurement error. BMJ.

[24] Andersson JL, Hutton C, Ashburner J, Turner R, Friston K (2001). Modeling geometric deformations in EPI time series. Neuroimage.

[25] Friston KJ, Holmes AP, Worsley KJ, Poline JP, Frith CD, Frackowiak RSJ (1994). Statistical parametric maps in functional imaging: A general linear approach. Hum Brain Mapp.

[26] Zaehle T, Schmidt CF, Meyer M, Baumann S, Baltes C, Boesiger P et al. Comparison of “silent” clustered and sparse temporal fMRI acquisitions in tonal and speech perception tasks. Neuroimage. 2007;37(4):1195–204. http://www.sciencedirect.com/science/article/pii/S105381190700479X. 10.1016/j.neuroimage.2007.04.073 17644001

[27] Liem F, Lutz K, Luechinger R, Jäncke L, Meyer M (2012). Reducing the interval between volume acquisitions improves “Sparse” scanning protocols in event-related auditory fMRI. Brain Topogr.

[28] Slotnick SD, Moo LR, Segal JB, Hart Jr J. Distinct prefrontal cortex activity associated with item memory and source memory for visual shapes. Cogn Brain Res. 2003;17(1):75–82. http://dx.doi.org/10.1016/S0926-6410(03)00082-X. 10.1016/s0926-6410(03)00082-x 12763194

[29] Forman SD, Cohen JD, Fitzgerald M, Eddy WF, Mintun MA, Noll DC (1995). Improved assessment of significant activation in functional magnetic resonance imaging (fMRI): use of a cluster-size threshold. Magn Reson Med.

[30] Eickhoff SB, Stephan KE, Mohlberg H, Grefkes C, Fink GR, Amunts K et al. A new SPM toolbox for combining probabilistic cytoarchitectonic maps and functional imaging data. Neuroimage. 2005;25(4):1325–35. http://www.sciencedirect.com/science/article/pii/S105381190400792X. 10.1016/j.neuroimage.2004.12.034 15850749

[31] Geyer S, Ledberg A, Schleicher A, Kinomura S, Schormann T, Burgel U (1996). Two different areas within the primary motor cortex of man. Nature.

[32] Geyer S, Schleicher A, Zilles K (1999). Areas 3a, 3b, and 1 of human primary somatosensory cortex: 1. Microstructural organization and interindividual variability. Neuroimage.

[33] Geyer S, Schormann T, Mohlberg H, Zilles K (2000). Areas 3a, 3b, and 1 of human primary somatosensory cortex: 2. Spatial normalization to standard anatomical space. Neuroimage.

[34] Grefkes C, Geyer S, Schormann T, Roland P, Zilles K (2001). Human somatosensory area 2: observer-independent cytoarchitectonic mapping, interindividual variability, and population map. Neuroimage.

[35] Eickhoff SB, Schleicher A, Zilles K, Amunts K (2006). The human parietal operculum. I. Cytoarchitectonic mapping of subdivisions. Cereb Cortex.

[36] Eickhoff SB, Amunts K, Mohlberg H, Zilles K (2006). The human parietal operculum. II. Stereotaxic maps and correlation with functional imaging results. Cereb Cortex.

[37] Diedrichsen J, Balsters JH, Flavell J, Cussans E, Ramnani N (2009). A probabilistic MR atlas of the human cerebellum. Neuroimage.

[38] Tzourio-Mazoyer N, Landeau B, Papathanassiou D, Crivello F, Etard O, Delcroix N (2002). Automated anatomical labeling of activations in SPM using a macroscopic anatomical parcellation of the MNI MRI single-subject brain. Neuroimage.

[39] Maldjian JA, Laurienti PJ, Kraft RA, Burdette JH (2003). An automated method for neuroanatomic and cytoarchitectonic atlas-based interrogation of fMRI data sets. Neuroimage.

[40] Mehta JP, Verber MD, Wieser JA, Schmit BD, Schindler-Ivens SM (2012). The effect of movement rate and complexity on functional magnetic resonance signal change during pedaling. Motor Control.

[41] Christensen LO, Johannsen P, Sinkjaer T, Petersen N, Pyndt HS, Nielsen JB (2000). Cerebral activation during bicycle movements in man. Exp Brain Res.

[42] Kapreli E, Athanasopoulos S, Papathanasiou M, Van Hecke P, Strimpakos N, Gouliamos A (2006). Lateralization of brain activity during lower limb joints movement. An fMRI study. Neuroimage.

[43] Rombouts SA, Barkhof F, Hoogenraad FG, Sprenger M, Valk J, Scheltens P (1997). Test-retest analysis with functional MR of the activated area in the human visual cortex. Am J Neuroradiol.

[44] Dice LR (1945). Measures of the amount of ecologic association between species. Ecology.

[45] Sørensen T (1948). A method of establishing groups of equal amplitude in plant sociology based on similarity of species and its application to analyses of the vegetation on Danish commons. Biol Skr.

[46] Shrout PE, Fleiss JL (1979). Intraclass correlations: uses in assessing rater reliability. Psychol Bull.

[47] Winter DA. Kinematic and kinetic patterns in human gait: Variability and compensating effects. Hum Mov Sci. 1984;3(1–2):51–76. http://dx.doi.org/10.1016/0167-9457(84)90005-8.

[48] Masani K, Kouzaki M, Fukunaga T (2002). Variability of ground reaction forces during treadmill walking. J Appl Physiol.

[49] Martinez M, Villagra F, Loayza F, Vidorreta M, Arrondo G, Luis E (2014). MRI-compatible device for examining brain activation related to stepping. IEEE Trans Med Imaging.

[50] Rushworth MFS, Krams M, Passingham RE (2001). The attentional role of the left parietal cortex: the distinct lateralization and localization of motor attention in the human brain. J Cogn Neurosci.

[51] Floyer-Lea A, Matthews PM (2004). Changing brain networks for visuomotor control with increased movement automaticity. J Neurophysiol.

[52] Field AS, Yen Y-F, Burdette JH, Elster AD (2000). False cerebral activation on BOLD functional MR images: study of low-amplitude motion weakly correlated to stimulus. Am J Neuroradiol.

[53] Jezzard P, Balaban RS (1995). Correction for geometric distortion in echo planar images from B0 field variations. Magn Reson Med.

[54] Wei X, Yoo S-S, Dickey CC, Zou KH, Guttmann CRG, Panych LP. Functional MRI of auditory verbal working memory: long-term reproducibility analysis. Neuroimage. 2004;21(3):1000–8. http://dx.doi.org/10.1016/j.neuroimage.2003.10.039. 10.1016/j.neuroimage.2003.10.039 15006667

[55] Eaton KP, Szaflarski JP, Altaye M, Ball AL, Kissela BM, Banks C et al. Reliability of fMRI for studies of language in post-stroke aphasia subjects. Neuroimage. 2008;41(2):311–22. http://dx.doi.org/10.1016/j.neuroimage.2008.02.033. 10.1016/j.neuroimage.2008.02.033 PMC2474692 18411061

[56] Seto E, Sela G, McIlroy WE, Black SE, Staines WR, Bronskill MJ (2001). Quantifying head motion associated with motor tasks used in fMRI. Neuroimage.

[57] Huettel SA, Singerman JD, McCarthy G. The Effects of Aging upon the Hemodynamic Response Measured by Functional MRI. Neuroimage. 2001;13(1):161–75. http://dx.doi.org/10.1006/nimg.2000.0675. 10.1006/nimg.2000.0675 11133319

[58] D'Esposito M, Zarahn E, Aguirre GK, Rypma B (1999). The effect of normal aging on the coupling of neural activity to the bold hemodynamic response. Neuroimage.

[59] Maclaren J, Herbst M, Speck O, Zaitsev M (2012). Prospective motion correction in brain imaging: A review. Magn Reson Med.

